# Feasibility and appropriateness of recommended sorghum production technologies

**DOI:** 10.1186/2193-1801-3-453

**Published:** 2014-08-22

**Authors:** Rajendra R Chapke

**Affiliations:** Directorate of Sorghum Research, ICAR, Hyderabad, India

**Keywords:** Appropriateness, Extension system, Feasibility, Innovative attributes, Research system, Sorghum production technology

## Abstract

Several initiatives were taken by the Directorate of Sorghum Research and concerned organizations to disseminate promising sorghum technologies. However, many of them were not accepted by the farmers at desired level due to several reasons. Therefore, it was felt necessary to assess the feasibility and appropriateness of recommended technologies as perceived primarily by the research system and followed by the extension personnel. These steps were felt to be a forerunner to screen the recommended technologies for their dissemination. The documentation of the sorghum production technologies/practices for both rainy and post-rainy season was made. The perception of 50 researchers regarding the feasibility of these technologies elucidated that out of 21 documented technologies, six were having feasibility scores of > 4.5 and < 2.2, while the rest of the nine technologies had a medium feasibility score in between 2.0 and 4.5 on a feasibility continuum ranging from 1.0 (not feasible) to 5.0 (highly feasible). Out of these six assessed technologies, extension personnel have perceived three each of technologies as highly appropriate and feasible. Correlation of eight indicators of appropriateness with feasibility of technologies was significant whereas, relative advantage had no correlation. Five indicators of appropriateness namely, simplicity, observability, physical compatibility, production sustainability and cost together explained 96.63 per cent of the total variation in feasibility. It stated that the five indicators are contributing significantly in feasibility of sorghum production technologies. These need to be taken into consideration while developing and disseminating the technologies.

## Introduction

As the agricultural scenario is changing very fast, the priority areas of research, extension and farmers systems are sought desirable modifications. Besides, the socio-economic, psychological, communication and innovation decision related behaviour of subjects under investigation, several other researchable areas are fast coming up. There is no doubt that scientific way of farming and adoption of viable techniques will enable the farmers to match the pace with changing agricultural scenario. Sorghum (*Sorghum bicolor* L. Moench) has potential to assure food security to increasing population in harsh climate unlike fine cereals like, rice and wheat especially in semi-arid regions. Interventions of feasible technologies for making it more remunerative in competition with commercial and vegetable crops, is a time demand Roling (1988). Several promising technologies have been developed and demonstrated to the farmers’ fields by the sister organizations since last three-four decades to meet the diverse needs of the farmers. Several efforts have been made by the extension personnel to transfer the technology to the farmers in order to achieve an increase in production and productivity. Despite these concerted efforts, a large number of recommended technologies do not accepted by the farmers at desired level due to several reasons Chapke et al. (2013). They are either being adopted in truncated manner or not at all (Drost et al. 1996). In India, agro-technologies including sorghum production generated so far have been readily accepted by the resource-rich farmers but in the resource-poor areas like, dryland and rainfed agriculture encompassing millions of small and marginal farmers are away from accessibility of technological development (Das 1996). Therefore, in order to make them ease to get the benefits of improved farm technologies, it is essential to assess the technology from the point of view of appropriateness and overall feasibility (Watson et al. 2005). Generally, for any technology to be appropriate it needs to be simple, convincing, need-based, location specific, socially and economically acceptable and environment friendly leading to sustainability. Appropriateness of a technology is a prerequisite for its transfer and adoption Verma (2007)). Hence, an attempt has been made to analyse some recommended sorghum production technologies with respect to their overall feasibility and appropriateness as perceived by the personnel of the research and extension systems.

### Methodology

#### Sampling plan

The major sorghum growing states namely, Maharashtra, Karnataka, Madhya Pradesh, Rajasthan, Andhra Pradesh and Gujrat in India were selected for the study. Responses were taken from 50 randomly selected personnel members of the sorghum research system including Directorate of Sorghum Research (DSR) and All India Coordinated Sorghum Improvement Projects (AICSIPs), and 30 personnel members of the extension system following the questionnaire survey method.

#### Documentation of recommended sorghum production technologies

The glossary of recommended sorghum production technologies was prepared after the documentation on the basis of published reports and related literatures of the different organizations working on sorghum in major sorghum growing states of India.

#### Feasibility and appropriateness of technologies as perceived by the personnel of the research and extension system

The perception of research personnel with respect to the feasibility of documented recommended sorghum production technologies was assessed with the help of a feasibility questionnaire developed for the study. Responses were taken from 50 research personnel regarding their perception with respect to the feasibility of recommended sorghum production technologies. Responses were taken on a feasibility continuum ranging from 5.0 (highly feasible) to 1.0 (not feasible). Explanation for the non-feasibility/lower feasibility of technologies as perceived by the experts was also recorded. Feasibility refers to the suitability of a technology to be adopted in the farmers’ situation optioned as highly feasible by the research personnel, were further assessed with respect to their feasibility and appropriateness as perceived by the extension personnel. Feasibility was assessed on the above-mentioned feasibility continuum. Unlike research personnel, extension personnel, being grass root level workers, are supposed to be more familiar with the farming system as they maintain close contact with the farmers. Therefore, besides feasibility of technologies they were asked to perceive the appropriateness as well. Appropriateness of technology was assessed with respect to nine indicators selected on the basis of a review of literature and on already laid down criteria of attributes of innovation. The indicators of appropriateness were simplicity-complexity, relative advantage, observability, cost, profitability, physical compatibility, cultural compatibility, need and production sustainability. Responses were recorded for each selected recommended technology with respect to each of the indicators on a continuum ranging from 1 to 5, that is unfavourable (poor/low) to favourable (best/high). Indicators of appropriateness have been operationalised as follows. A total of 21 sorghum production technologies were selected on the basis of published documents of different organisations working on sorghum research in the country. The feasibility of all these technologies as perceived by different personnel of the research system was assessed. Primarily, the weighted mean score and standard deviation were derived for each technology on the basis of responses of all 50 personnel members of the research system. It was thought to be a crude method as the weighted mean scores might have been affected due to feared or biased responses of a few of the personnel. Therefore, further statistical analyses were carried out to check such biases and elimination of such personnel. For this, a correlation analysis was done and a correlation matrix was formed, on the basis of which the degree of agreement with respect to the responses of each of all the research personnel with others was found highly significant at a five per cent level of significance. Frequency of agreement of each personnel members with others and standard deviation were derived. Finally, it was found that no research personnel having negative correlation with the others and had significant disagreement with others as well. Thus, all the personnel of the research system were considered for the analysis and on the basis of their responses, mean feasibility score and standard deviation for each technology was worked out.

### Attributes of innovation

#### Simplicity-complexity

The simplicity dimension of the technologies is referred as the degree to which a technology is easy to understand, operate and use, whereas, complexity refers to the degree to which a technology is difficult to use and understand.

### Relative advantage

The degree to which the technology is perceived as better than the idea it supersedes.

### Observability

The degree to which the result of adoption of a technology is visible. The visible impact of a technology facilitates its diffusion.

### Cost of the technology

It refers to the investments involved in its purchase plus the recurring cash expenses on it and cash expenses on other associated activities necessary for putting the practice into operation.

### Profitability

Profitability of each technology is reffere monetary and physical returns obtained by adopting the technology as compared to that one it substitutes.

### Physical compatibility

Is the degree to which a technology (s) is in conformity with the existing situation of the farming community. In other words, physical compatibility refers to how well a practice fits into the working conditions of farmers.

### Cultural compatibility

Is the degree to which a technology is consistent with the existing beliefs, values, attitudes, living patterns, habits, cultural norms and past experiences of the farmers. For the purpose of this study, it refers to what extent a technology is compatible with the existing norms, values, and beliefs, past experiences of the respondents.

### Need of the technology

Need of the technology is referred to as the farmers’ perception of the requirements as well as cruciality of the technology in their setting.

### Production sustainability

Refers to the successful management of resources that maintain the quality of environment without any deterioration of the farmer’s production system.

## Findings and discussion

### Feasibility of technologies as perceived by the research personnel

The analysis on the basis of research personnel’s responses against total 21 selected sorghum production technologies, mean feasibility score and standard deviation for each technology was worked out.

It was found that six each technologies had a feasibility score of > 4.5 and < 2.2, while the rest of the nine technologies had a mean feasibility score in between 2.0 and 4.5 on a feasibility continuum ranging from 1.0 (not feasible) to 5.0 (highly feasible), where 3.0 indicates the neutral point. Further, categorization of the technologies on the basis of responses was made with the help of pooled standard deviation and pooled mean values, as indicated in Table 1. It was observed that six technologies each were perceived as highly feasible as well as low feasible. The rest of the nine technologies were found to be under the medium category.

**Table 1:** **Overall feasibility of 21 recommended sorghum production technologies as perceived by the personnel of the research system**

Sl. no.	Feasibility categories	Score	No. of technologies	Pooled mean	Pooled standard deviation
1	High	≥ 4.54	06	3.38	1.16
2	Medium	2.23-4.53	09		
3	Low	≤ 2.22	06		

The explanations of non-feasibility/lower feasibility of six technologies (mean feasibility score < 2.2) as mentioned by the respondents have been indicated in Table 2. It is revealing that there are several perceived constraints, which may be responsible for the non-feasibility/lower feasibility of technologies in the farmers’ system. Use of technologies is required additional labourer and costs which is not or very less cost-effective for this sorghum crop. Farmers do not like to adopt technologies that do not show any visible increase in monetary benefits. The technologies like, (i) use of organic fertilizers (farm yard manure or vermi-compost), (ii) chemical seed treatment, (iii) seed treatment with bio-agent (azotobactor), (iv) manual weed control, (v) use of four irrigations and (vi) soil application of pesticides, suffer from non-adoption in the farmers’ system due to its costly affair. Rathod et al. 2013 also reported in the similar line that low adoption was recorded due to non availability and cost of inputs, poor quality and physical properties of bio-fertilizers. It is perceived to be difficult to these six technologies in the sorghum areas where farmers’ priority was commercial crops.

**Table 2:** **Reasons for non feasibility/lower feasibility of some recommended sorghum production technologies/practices as perceived by the personnel of the research systems**

Sl. no	Recommended technologies/practices	Perceived reasons for non feasibility/lower feasibility of the technology/practice in the farmers’
1	Use of organic manure (farm yard manure @10 t/ha or vermi-compost @ 7-8 t/ha at last ploughing)	FYM and vermicompost are not available in sufficient quantity. These are not economical to use in sorghum due to their high costs.
2	Seed treatment with14 ml Imidacloprid (*Goucho*) + 2 g Carbendazim (*Bavistin*) per kg of seed or Thiomethaxam (*Cruser*) 3 g per kg of seed.	Seed treatment with the recommended chemicals is not practical in farmer’s fields. These are adding cost also.
3	Seed treatment with bio-fertilizer (Azotobactor at 250 g/10 kg seed)	Availability of Azotobactor at grass root level is very difficult. Proper handling and use of bio-agents is technically uncomfortable.
4	Weed control manually 2-3 times at 15 days interval after 25-30 days of emergence	It requires more labourer. High wages of labourer and their shortage in peak season is serious problem.
5	Use of four irrigations (each irrigation at panicle initiation, boot leaf, flowering and grain filling stages)	There is scarcity of irrigation water in almost all the sorghum areas. If available, it will be used for other commercial or vegetable crops
6	Soil application of Carbofuron 3G @ 2 g/m at sowing to control insect-pests	It is not a regular practice and need laborer. It can again add cost which is not economical.

It is evident that six out of 21 recommended technologies were screened as highly feasible by the research personnel. Jain et al. 1993 reported that it was essential to know which technologies were suitable or likely to be adopted by the farmers. It is in this context that prioritizing of technologies by personnel of the research system is the first step in the technology assessment process. In the present study, research personnel could anticipate several reasons for lower feasibility of some recommended sorghum production: labour intensive, costly, practically difficult and less remunerative. According to Patil et al. 2013 researchers have developed basket of sorghum technologies to find solutions to the problems relating to food and nutritional security, however, experiences have shown that in a large percentage of cases, feasibility is bottlenecked by factors such as high laboure requirement, high operational costs, accessibility of inputs and marketing.

### Feasibility and appropriateness of technologies as perceived by the extension personnel

The extension personnel are supposed to maintain the linkage and feedback mechanism between research and the farmers’ system. As the technology dissemination process, it was thought proper to ask them to anticipate the appropriateness besides the overall feasibility with respect to 21 recommended sorghum production technologies. Primarily, the weighted mean score and standard deviation were derived for each technology on the basis of the responses of 30 extension personnel. The categorization of the technologies on the basis of responses was made with the help of pooled standard deviation and pooled mean values in both cases as indicated in Table 3. It was observed that three of each technologies were perceived as highly feasible and appropriate. While three and four technologies were found to be perceived as low feasible and appropriate, respectively. The rest of the technologies falls under the medium category. An account of the technologies, perceived as highly feasible and appropriate is presented in Table 4.

**Table 3:** **Feasibility and appropriateness of the technologies as perceived by the personnel of the extension system**

Sl. no.	Feasibility categories	Score	No. of technologies	Pooled mean	Pooled standard deviation
1	High	≥ 4.40	03	4.13	0.27
2	Medium	3.87-4.39	15		
3	Low	≤ 3.86	03		
**Sl. no.**	**Appropriateness categories**	**Score**	**No. of technologies**	**Pooled mean**	**Pooled standard deviation**
1	High	≥ 4.22	03	4.08	0.14
2	Medium	3.95-4.21	14		
3	Low	≤ 3.94	04

**Table 4:** **Recommended sorghum production technologies perceived as highly feasible and appropriate by the personnel of both the research and extension systems**

Sl. recommended		Research personnel		Extension personnel			
No technologies/practices		Mean feasibility score	Standard deviation	Mean feasibility score	Standard deviation	Mean appropriateness score	Standard deviation
1	Land preparation (one ploughing, 2-3 harrowings, stubbles picking)	4.72	0.45	4.57	0.50	4.20	0.30
2.	Use of high yieldingvariety/hybrid	4.08	0.94	4.27	1.00	4.30	0.29
3.	Sowing time between 2^nd^ week of September and 2^nd^ week of October for *rabi* sorghum	4.00	1.20	4.53	0.62	4.25	0.40
4.	Use of seeds @8-10 kg/ha (3-4 kg/acre)	4.60	0.49	4.57	0.50	4.29	0.27
5.	Spacing between lines 45 cm and between plants 12-15 cm	4.56	0.50	4.30	0.78	4.13	0.37
6.	Inter-cultivation 2-3 times at 3,5 and 7 weeks after emergence	4.60	0.61	3.73	1.09	4.02	0.48
7.	Limited irrigations (should be given at boot leaf or flowering stage)	4.60	0.49	4.23	0.92	4.19	0.33
8.	Harvesting at physiological maturity	4.60	0.64	4.37	0.80	4.16	0.49

**Figure 1:**
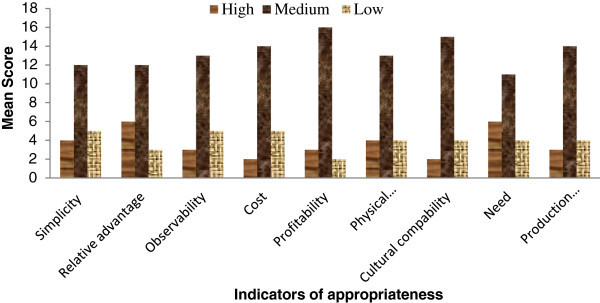
**Indicators of appropriateness of sorghum production technologies perceived by extension personnel.**

The overall appropriateness as mentioned above was derived on the basis of responses on nine innovative attributes. Perceptions of extension personnel with respect to all 21 technologies on different indicators of appropriateness are depicted in Figure 1. It is evident that few technologies were perceived as low with respect to indicators like, profitability, relative advantage, cultural compatibility and production sustainability. In contrast, some technologies were perceived highly with respect to different indicators. Refinement with respect to respective indicators on a technology perceived as low will improve the overall appropriateness of that technology. A correlation of different indicators of appropriateness with feasibility of technologies was worked out. It is revealing that eight out of nine indicators of appropriateness were significantly related to feasibility (Table 5). Hoverer, cost has been related highly negative, prompting that costly sorghum technologies are not feasible to use. Chapke et al. (2013) and Ofuoku et al. (2008), confirmed in the same line that high cost and complexity of the technology are the major bottlenecks in the adoption. The zero order correlation indicated the relationship of one independent variable at a time with the dependent variable and did not indicate the intensity of the relationship. The data was therefore put to step-wise multiple regression analysis considering nine indicators of appropriateness as independent variables and feasibility as a dependent variable. The result is presented in Table 6. The step-wise regression went up to three steps and finally, five indicators of appropriateness: simplicity, observability, physical compatibility, production sustainability and cost were appeared in the optimum regression model (step III). These together explained 96.63 per cent of the total variation in feasibility with the ‘t’ values being significant. It means that the four omitted variables during I^st^ and II^nd^ steps jointly contributed to only 3.37% per cent of non-significant variation. Based-on these findings it may be concluded that the five indicators are contributing significantly in feasibility of sorghum production technologies. This finding also indicated that these attributes are very important while selecting the technologies for refining and advocating to the farmers. Some supportive measures like, availability of inputs and regularized market need to be considered as per the different locations. It is revealing that extension personnel have perceived three technologies as highly feasible out of six recommended technologies already assessed highly feasible by the research personnel. It may be attributed to the differential perception of the criteria for feasibility of technologies. In principle, the standards of farmers and extension and research personnel are complementary, but in practice their formal expression and purpose of application differ considerably (Schiere and de Wit 1993; Singh 1996). The perception with respect to the appropriateness of technology was indicated on nine indicators of appropriateness out of which simplicity, observability, physical compatibility, cultural compatibility, and cost together contributed to 96.63 per cent of the total variation in feasibility. Many studies have used criteria for appropriateness of technology such as adaptability, profitability, economic viability, observability, simplicity, cultural compatibility, extent of risk, need based, and sustainability (Jain et al. 1993; Singh and Schiere 1994; Choudhary 1995). Chapke 2001 reported that simplicity, congruity and profitability of farm technologies had direct correlation with adoption. Although extension personnel perceived only three technologies as highly appropriate, and also three technologies as highly feasible. Therefore, it may be concluded that not only the appropriateness but also feasibility of technologies have vital role in adoption process.

**Table 5:** **Correlation of different indicators of appropriateness with feasibility of technologies**

Sl. no.	Indicators of appropriateness	Correlation coefficient (r)
1	Simplicity	0.690**
2	Relative advantage	0.413
3	Observability	0.536**
4	Cost	062*
5	Profitability	0.328*
6	Physical compatibility	0.940**
7	Cultural compatibility	0.734**
8	Need	0.763**
9	Production sustainability	0.677**
10	Overall appropriateness	0.855**

**Significant at 1% level, *Significant at 5% level.

**Table 6:** **Stepwise regression analyses between feasibility (dependent variable) and indicators of appropriateness (independent variables)**

Variables	‘b’	val ‘t’	val ‘P’	val R ^2^
**Step I**				
Simplicity	0.12873	1.40	0.1839	0.9685
Observability	−0.18122	−2.99	0.0105	
Physical compatibility	0.57450	8.70	0.0000	
Cultural compatibility	0.01298	0.15	0.8828	
Need	−0.14108	−0.95	0.3587	
Production sustainability	0.32268	2.79	0.0154	
Cost	−0.25440	−3.21	0.0069	
**Step II**				
Simplicity	0.13259	1.56	0.1408	0.9684
Observability	−0.18348	−3.24	0.0059	
Physical compatibility	0.57468	9.03	0.0000	
Need	−0.13664	−0.98	0.3461	
Production sustainability	0.33337	3.79	0.0020	
Cost	−0.25550	−3.36	0.0047	
**Step III**				
Simplicity	0.14217	1.69	0.1121	0.9663
Observability	−0.18162	−3.21	0.0058	
Physical compatibility	0.54338	9.90	0.0000	
Production sustainability	0.31796	3.68	0.0022	
Cost	−0.21539	−3.37	0.0042	

## Conclusion

Technology transfer is a vital but complex process in agricultural and rural development. In order to available resources, adoption of improved production technologies is one of the major options for sorghum farmers to raise their livelihood. Over the years, technology generation by the researchers and dissemination by extension personnel have been carried out to meet the requirement of the farmers as food, feed and fodder. However, the majority of the recommended technologies do not find place in farmers’ system. It is, therefore, necessary to prioritize the technologies with respect to their feasibility and appropriateness before their dissemination to the farmers’ system. In this context, technology assessment primarily as perceived by the research personnel, then by the extension personnel is prime importance. Since every technology cannot be graded as neutral, it is very necessary to analyse the existing technologies for their appropriateness in the specific agro-climatic farming condition. It is also necessary to refine or modify the recommended existing technologies to make them viable and suitable according to the needs and resources of farmers of a specific farming system. Therefore, it will be advisable to carry out further assessment of the technologies in the location-specific situation, already perceived highly feasible and appropriate by both personnel of the research and the extension system in this study. Simultaneously, studies on factors responsible for limiting the process of knowledge exchange and adoption and delineation of parameters on the basis of in-appropriateness of the technologies need to be done. It will provide feedback for personnel of research and extension systems for revitalizing the technology generation and effective dissemination system.
